# Presentation of Rarely Occurring Inverted Squamous Papilloma of Nasal Cavity: A Case Report

**DOI:** 10.7759/cureus.62461

**Published:** 2024-06-16

**Authors:** Amit Bhoyar, Ashish Anjankar, K. M. Hiwale, Suhit Naseri

**Affiliations:** 1 Department of Pathology, Jawaharlal Nehru Medical College, Datta Meghe Institute of Higher Education and Research, Wardha, IND; 2 Department of Biochemistry, Jawaharlal Nehru Medical College, Datta Meghe Institute of Higher Education and Research, Wardha, IND

**Keywords:** histopathology, surgical resection, tumor, sinus cancer, biopsy, sinonasal tract

## Abstract

This report presents a case of a rarely occurring inverted squamous papilloma, which shows papillary proliferation in squamous epithelium. Inverted papillomas (IPs) are benign epithelial growths that occur in the underlying stroma of the nasal cavity and paranasal sinuses. While viral infections, allergies, and chronic sinusitis have all been proposed as potential causes, the pathophysiology of this lesion is still unknown. Most of the time, IP reflects residual disease, yet the recurrence rates are extremely high. A 60-year-old male patient has chief complaints of right-sided nasal congestion and excessive sneezing and discharge from the nose, which cause discomfort to the patient and make him unable to sleep at night. Computed tomography reveals both the enlargement of the osteomeatal complex and soft-tissue density opacification of the right side of the nasal cavity. The only way to diagnose this type of squamous papilloma is through histopathological examination. In this work, we evaluated the histological characteristics of sinonasal IP and presented a case report of an uncommon instance of inverted squamous papilloma of the nasal cavity.

## Introduction

The cancer mortality rate in India experienced a more than twofold increase between 1990 and 2016. India's cancer incidence is predicted to have reached 1.15 million new cases in 2018 and, as a result of changes in the country's population, is predicted to nearly triple by 2040 [1]. The sinonasal area includes both the nasal cavity and the paranasal sinuses. Sinonasal cavities are tiny anatomical spaces that are inhabited by a wide array of tumors with varying histologies. The nose and paranasal sinuses are the source of epithelial and nonepithelial neoplasms. Olfactory mucosa, tiny salivary glands, or mucosa originate from epithelium-related malignancies. Mesenchymal tissue in the sinusoidal tract imparts its conception to malignant cells [2].

A very diverse subset of tumors, sinonasal tumors make up approximately 3% of head and neck cancers and less than 1% of all malignancies. Different cell types, such as epithelial cells, glandular, and mesenchymal cells, can give rise to these tumors. Despite being uncommon, sinonasal malignancies pose a serious clinical risk because of their aggressive nature and difficult diagnosis [3]. Nasal cavity cancer is frequently only suspected secondary to other more common illnesses, such as chronic rhinitis or sinusitis, which frequently have similar symptoms. Exposure to nickel, wood dust, and chemical solvents is a risk factor for cancer of the nasal cavity. Cigarette smoking is also substantially implicated [4].

Inverted papilloma (IP) often originates from the nasal cavity's lateral wall. They can locally expand to the paranasal sinuses and even the nasopharynx. On rare occasions, they might move over the cribriform plate or orbit, especially if they are linked to cancer. They rarely emerge from the nasal septum [5]. An IP is the most common benign tumor found in the nasal epithelium and sinus cavities. Surgical intervention alone has been the primary treatment for IPs, with clinical follow-up in between has occurred on occasion. It is clinically critical to rule out squamous cell carcinoma (SCC), the most prevalent tumor among malignancies of the nasal and sinonasal cavities, when IP is suspected in the lesion [6]. Sinonasal cavities include a histopathologically heterogeneous collection of masses in a small, complex space. A wide range of neoplastic and nonneoplastic diseases are observed in ordinary practice. Polypoidal tumors are the most frequent lesions in the nasal cavity [7].

Computed tomography (CT) clinically indicates that invasive soft-tissue lesions in the paranasal sinuses are distinguished by their nonhomogeneous structure. These tumors can potentially demolish the sinus's bone borders, invade adjacent regions in certain directions, and become more visible after contrast is administered [8]. The sinonasal tract is a unique location for tumor formation because nearly any kind of tumor can occur there. Based on clinical observations, modern radiological techniques, and patient symptoms, most clinicians make an assumption diagnosis. Histopathological examination (HPE) is still the preferred method of diagnosis. Precise HPE can significantly impact the course of treatment and follow-up for some disorders, particularly in rare conditions resembling typical benign conditions [9].

## Case presentation

We report the case of a male patient, 60 years old, who was taken to the hospital complaining of nasal obstruction and nasal discharge. The patient was fine seven months ago when he began experiencing nasal discharge. The nasal discharge was insidious in onset, whitish in color, nonfoul smelling, scanty in amount, and aggravated during the nighttime association with sneezing, due to which the patient’s sleep was disturbed. The general examination of the patient is normal, and all regular tests have been completed. Laboratory reports state that all of the results were normal, as shown in Table 1.

**Table 1:** Laboratory results and reference ranges PT: prothrombin time; APTT: activated partial thromboplastin time; RBS: random blood sugar; IU: international unit

Investigation	Observed value	Reference range
Hb %	12.7 g/dL	Male: 13-17 g/dL; female: 12-15 g/dL
RBC count	5.53 million/μL	Male: 4.5-5.5 million/μL; female: 3.8-4.8 million/μL
WBC count	7,600/μL	Male and female: 4,000-10,000/μL
Total platelet count	2.28 (IU/μL)	Male and female: 150,000-400,000 (IU/μL)
PT	11.9 seconds	10-12 seconds
APTT	33 seconds	25-38 seconds
Sodium	142 mEq/L	135-145 mEq/L
Potassium	5.2 mEq/L	3.5- 5.2 mEq/L
RBS	82 mg/dL	70-110 mg/dL
Creatinine	3.9 mg/dL	0.7-1.3 mg/dL

**Figure 1:**
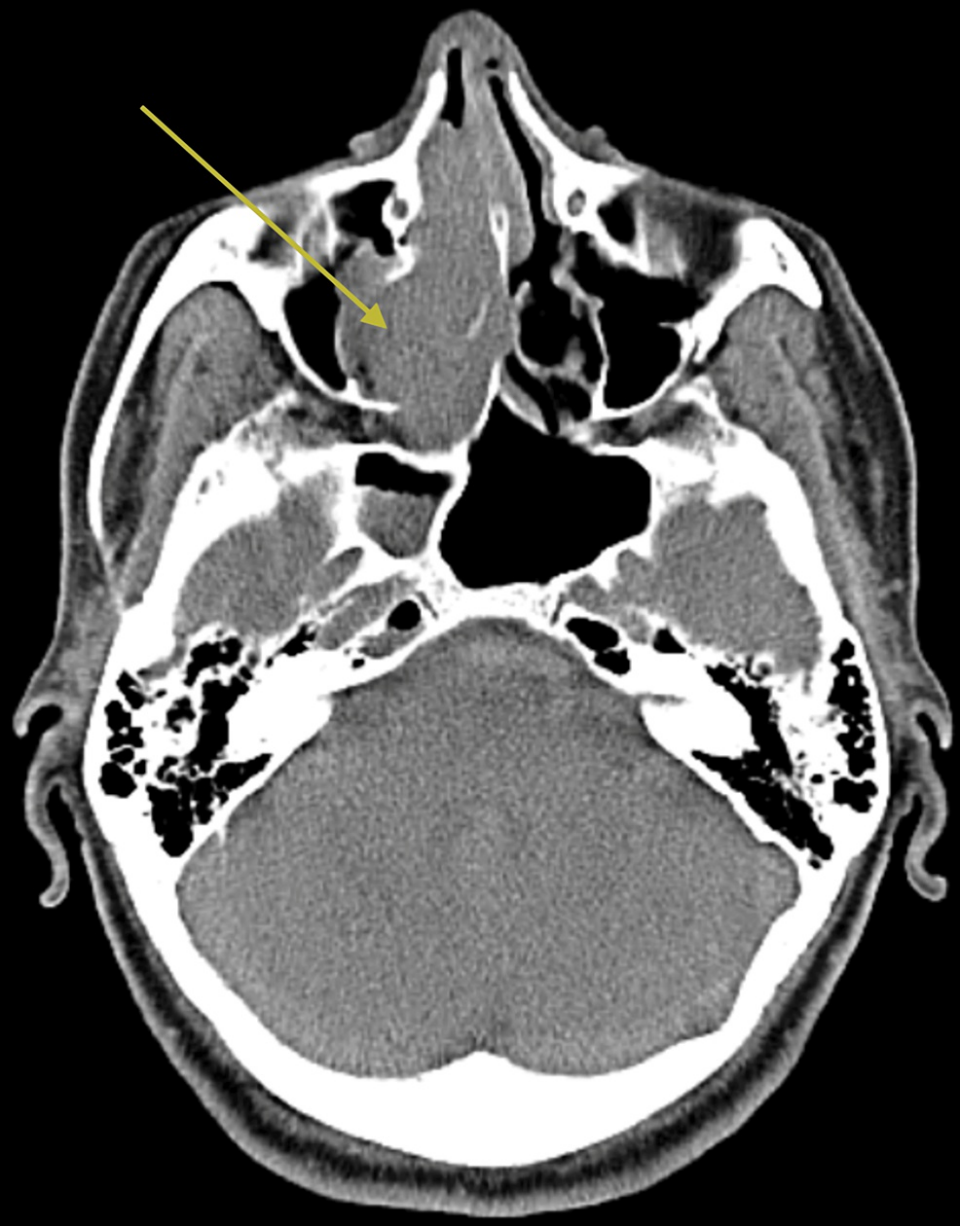
CT showing the soft-tissue density of the right side of the nasal cavity and also the widening of the osteomeatal complex (yellow arrow) CT: computed tomography

The CT scan reveals soft tissue density in the right side of the nasal cavity, including the anterior and posterior ethmoidal air cells, which extend beyond the right posterior choanae and reach up to the nasopharynx, causing expansion of the right nasal cavity over a length of approximately 1.3 cm beyond the posterior choana. There is also an expansion of the right osteomeatal complex with its involvement, but no visible lesion is seen in the right maxillary sinus cavity (Figure 1).

**Figure 2:**
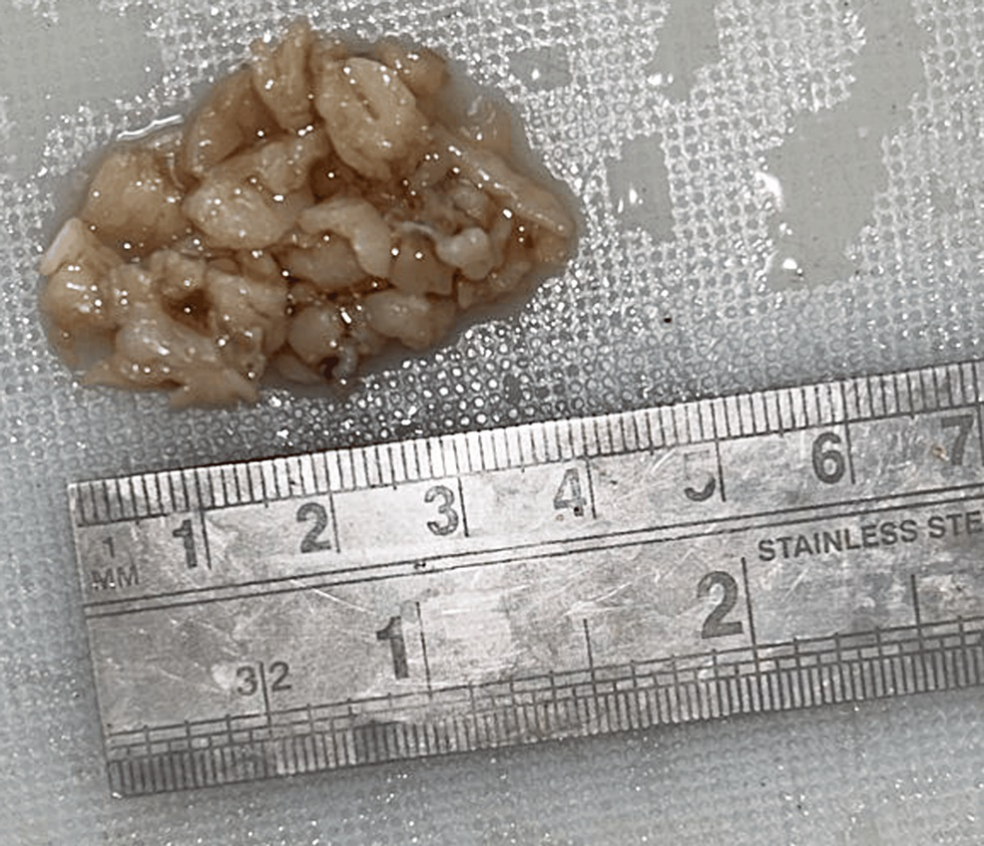
The gross image of the resected tumor (measuring 4 × 2 × 1 cm)

During the procedure under general anesthesia, the bilateral nasal cavities were packed with 2% xylocaine and adrenaline for 10 minutes, and then removed. A diagnostic nasal endoscopy was done. A biopsy was taken from both sides, and samples were sent for HPE. Hemostasis was achieved. For histological assessment, the specimen was received in a container labeled as a mass from the right nasal cavity. It consisted of whitish-gray tissue pieces measuring 4 × 2 × 1 cm, as illustrated in Figure 2.

**Figure 3:**
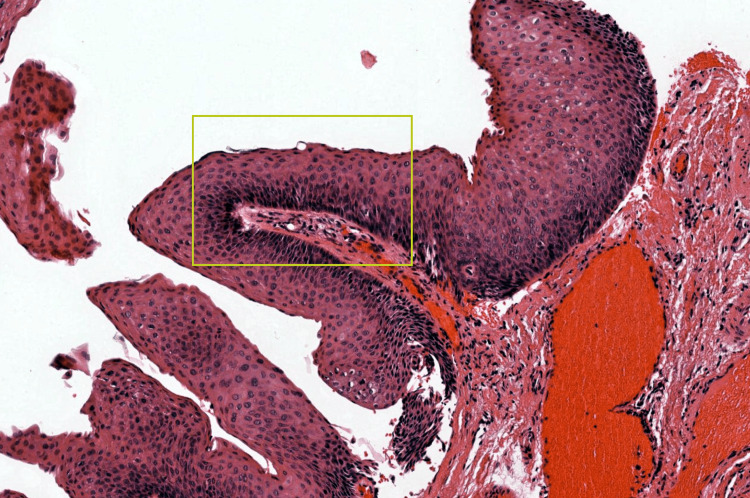
Hematoxylin and eosin staining (10×) section showing papillary proliferation covered by squamous epithelium (yellow box)

Operative histopathology revealed the results of a Hematoxylin and Eosin staining (10×) section showing papillary proliferation covered by squamous epithelium (yellow box). Histopathological features suggestive of inverted squamous papilloma of the nasal cavity are shown in Figure 3.

The patient underwent histology tests to clarify the disease and was advised to remain in the hospital for endonasal surgery (Table 2). The patient has been authorized to adhere to the dietician's entire diet recommendations. The patient's vital signs were stable, and, as recommended, the patient took the medication as per the treatment plan outlined in Table 3. After that, the patient was permitted to be discharged from the hospital.

**Table 2:** Treatment in the hospital OD: once daily; BD: twice daily

Medicine	Quantity	Dose	Days
Tablet sodium bicarbonate (500 mg)	8	OD	8
Tablet alfacalcidol (0.25 mg) + calcium carbonate (500.0 mg)	4	OD	4
Multivitamin and multimineral syrup	3	BD	6

**Table 3:** Prescription on discharge BD: twice daily; TDS: thrice daily; HS: at bedtime; OD: once daily; N/D: nasal drop

Medicine	Quantity	Dose	Days
Tablet amoxicillin (500 mg) + clavulanic acid (125 mg)	10	BD	5
Tablet rabeprazole (20 mg)	10	BD	5
Tablet trypsin + chymotrypsin	15	TDS	5
Tablet levocetirizine (5 mg) + ambroxol (75 mg)	5	HS	5
Tablet vitamin C (500 mg)	5	OD	5
Tablet sodium bicarbonate (500 mg)	15	TDS	5
N/D nasoclear 0.65% nasal spray (sodium chloride IP 0.65% w/v in distilled water + benzalkonium chloride solution IP 0.03% w/v)	1	2 drops TDS	5

## Discussion

According to studies in India, the country experiences approximately one case of sinonasal malignancies per 100,000 people annually. The most common histological subtypes of sinonasal malignancy in India are adenocarcinoma and SCC. Together, these two kinds account for most instances; SCC accounts for around 50% of sinonasal tumors, while adenocarcinoma accounts for the remaining 13% [3]. Bosch et al. conducted a study on the incidence of cancers in the nasal cavity and found that the incidence is higher than that of the ethmoidal, sphenoidal, or frontal sinuses and second only to the maxillary antrum. This is one of the reasons why nasal cavity tumors should be assessed separately. When paranasal sinus tumors reach a late stage and tend to enter the nasal fossa, pinpointing the exact location of origin might be challenging [10].

Harbo et al. demonstrated that univariate analysis has indicated a range of prognostic criteria for malignant tumors in the paranasal sinuses and nasal cavities because these tumors are extremely rare. Several different histology tumor types exist in the paranasal sinuses and nasal cavity. SCC (46%), in comparison to other head and neck cancer sites, is less common. Studies show significant differences in the relative distribution of adenocarcinoma and undifferentiated carcinoma [11]. Shirazi et al. discovered that less than 1% of all cancers are sinonasal tumors, a very diverse collection of tumors. In almost all cases, magnetic resonance imaging and CT scans were performed to evaluate the degree of malignancy and bone deterioration. The most common reason for treatment failure was local recurrence. Fungi-induced sinusitis was the most frequent cause of this. According to the study's findings, SCC is the most prevalent cancer in this area. Because of their proximity to important tissues, these tumors present considerable obstacles to therapy and may be the cause of significant morbidity in patients; thus, they require accurate and thorough research [12].

Segal et al. demonstrated that IPs are uncommon tumors, accounting for between 0.5% and 4% of all primary nasal malignancies. The fifth and seventh decades have a far higher incidence of IPs. It is well known that IPs and cancer are associated. IPs are always treated surgically. On the other hand, ethmoidectomy through lateral rhinotomy and medial maxillectomy significantly decreased the recurrence rate. This method of treating IPs is, in our opinion, the preferred surgical procedure [13]. Tatekawa et al. discovered that CT could reveal the origin of IPs, which are frequently associated with local hyperostosis on the nasal wall. Although this study concluded that bone sclerosis is more widespread among various inflammatory sinonasal disorders [14], Sharma et al. researched a range of inflammatory, nonneoplastic, and cancerous masses that are frequently seen in clinics that affect the nasopharynx, paranasal sinuses, and nasal cavity. It assesses the relationship between the histological diagnosis and the clinical and radiological findings. Fifty cases of masses in the nasopharynx, paranasal sinuses, and nasal cavity were analyzed. SCC was the most frequently found malignant lesion among the neoplastic lesions under investigation, while IPs were the most frequently found benign lesions. This study found that while histology always offers a confirmed diagnosis, radiography serves as a guide for endoscopic surgeons regarding any current or potential problems [15].

## Conclusions

This case study highlights the unusual occurrence of an inverted squamous papilloma in a male 60-year-old nasal cavity, which indicates papillary proliferation coated in squamous epithelium. In clinical practice, a variety of common and unusual disorders can manifest as nose and paranasal tumors. Our study demonstrates that HPE testing can effectively discriminate between patients with similar clinical complaints but separate underlying illnesses. CT provides information on the correct origin of IP, which is typically linked to localized hyperostosis on the nasal wall. It also displays malignant soft tumor tissue, which is distinguished by its nonhomogeneous structures. Tumor identification and proper surgical resection were achieved using examination, imaging tests, and histological analysis. Although inverted sinonasal papillomas are typically benign, they can exhibit locally aggressive behavior and damage nearby structures. They only make up 0.5%-4% of primary nasal tumors. IPs can be suspected when a mass in the nasal cavity appears to be emerging from the lateral nasal wall, affects one or more paranasal sinuses, and develops in a male patient in his fifth or sixth decade of life. The aggressiveness and rarity of the inverted nasosinusal papilloma contribute to the significance of this case. Understanding this unusual presentation and its possible consequences is essential for proper diagnosis, treatment, and long-term monitoring of individuals with sinonasal papilloma. Because of this, the surgical approach for this kind of uncommon IP must be carefully planned to improve the patient's quality of life.
